# Counteracting methicillin resistant *Staphylococcus aureus* through novel Citral-Azithromycin combination

**DOI:** 10.1038/s41598-025-11721-4

**Published:** 2025-07-15

**Authors:** Hitesh K. Sharma, Ibha Singh, Amarnath Karna, Puneet Gupta, Taru Singh, Anoop Kumar, Deepti Pandita, Monalisa Mukherjee, Virinder S. Parmar, Pallavi Agarwal, Viney Lather

**Affiliations:** 1 Amity Institute of Pharmacy, Amity University Uttar Pradesh, Sector 125, Noida, 201313 India; 2 Amity Institute of Molecular Medicine and Stem Cell Research, Amity University, Uttar Pradesh, Sector 125, Noida, 201313 India; 3 Amity Institute of Biotechnology, Amity University, Sector 125, Noida, 201313 India; 4 Department of Pharmacology, Delhi Pharmacological Sciences and Research University (DPSRU), New Delhi, 110017 India; 5 Department of Pharmaceutics, Delhi Institute of Pharmaceutical Sciences & Research (DIPSAR) Delhi Pharmaceutical Sciences and Research University, New Delhi, India; 6 Government of NCT of Delhi, New Delhi, 110017 India; 7 Amity Institute of Click Chemistry and Research Studies, Amity University Uttar Pradesh, Sector 125, Noida, 201313 India; 8 Nanoscience Program, CUNY Graduate Center, Department of Chemistry and Environmental Science, Medgar Evers College, The City University of New York, 1638 Bedford Avenue, 10025 NY New York, USA

**Keywords:** Antimicrobials, Infectious diseases

## Abstract

Methicillin-resistant *Staphylococcus aureus* (MRSA) poses a significant challenge, necessitating novel therapeutic strategies. This study demonstrates the synergistic bactericidal activity of citral, a phytochemical, and azithromycin against clinical MRSA isolates. Checkerboard assays confirmed synergy with a fractional inhibitory concentration index (FICI) of 0.093, while time-kill kinetics showed a reduction in bacterial viability from 5.39 to 2.94 log_10_ CFU/ml, outperforming monotherapy. Scanning electron microscopy revealed severe cellular damage, indicative of membrane disruption and protein leakage. Notably, the combination extended post-antibiotic effects (PAE) by 4.26 h at MIC, suggesting prolonged antibacterial action. Cytotoxicity assays on normal human fibroblasts demonstrated 93.7% viability at the combined MIC, underscoring its biocompatibility. These findings highlight citral-azithromycin synergy as a promising strategy to enhance antibiotic efficacy against MRSA while minimizing cytotoxicity, supporting the potential of phytochemical-adjuvant combinations in combating antimicrobial resistance.

**Supplementary Information:**

The online version contains supplementary material available at 10.1038/s41598-025-11721-4.

## Introduction

Antibiotic-resistant bacterial infections pose a severe and escalating global health threat, necessitating innovative and effective therapeutic strategies to counteract the rapid emergence of multidrug resistance (MDR)^1–3^. Among these pathogens, *Staphylococcus aureus* (*S. aureus*) stands out as a leading cause of healthcare-associated infections, exhibiting an extraordinary ability to acquire resistance through various mechanisms^4^. These include horizontal gene transfer, target modification, efflux pump overexpression, and biofilm formation, significantly complicating treatment strategies^3,5,6^. Notably, *S. aureus* not only contributes significantly to mortality associated with antibiotic resistance but also ranks among the leading causes of healthcare-associated infections^7^. The adaptability of *S. aureus* to develop resistance to antibiotics through diverse mechanisms, including the acquisition of resistance genes, target modifications, efflux pumps, and biofilm formation, exacerbates the challenge of combating these infections^8^.

The growing incidence of MDR infections emphasizes the urgent need to address the emergence of highly resistant forms of *S. aureus*, including methicillin-resistant (MRSA), vancomycin-intermediate (VISA), and vancomycin-resistant (VRSA) strains. Exposure of MRSA to antibiotics initiates a cascade of events leading to the development of drug resistance^9,10^.

The lag in antibiotic discovery and development highlights the necessity of exploring alternative strategies to combat antimicrobial resistance in bacterial populations^1,11,12^. Consequently, alternative methodologies like the development of synergistic antibiotic-phytomolecule combinations can be essential for effectively managing resistance and addressing severe diseases^13,14^. One promising approach is combination therapy, which involves synergistically pairing antibiotics with phytochemicals or other bioactive molecules to enhance bacterial susceptibility, inhibit resistance mechanisms, and minimize drug toxicity^15^. Such combinations offer synergistic effects by targeting multiple sites, enhancing pharmacokinetic properties, disrupting resistance mechanisms, and eliminating toxic components, thereby bolstering therapeutic efficacy^15–17^.

Plants serve as an invaluable reservoir of unique chemicals, offering promising avenues for the development of novel therapeutic options against resistant pathogens^18–20^. Many phytomolecules possess various pharmacological characteristics and have a long history of being used safely^21–23^. Plants synthesize a diverse array of secondary metabolites, including alkaloids, glycosides, flavonoids, and terpenoids, among others. These bioactive compounds exhibit a broad spectrum of pharmacological activities, such as antibacterial, anticancer, anti-inflammatory, and analgesic properties^24,25^. Exploring plant-based substitutes for antibiotics has considerable potential, and the enthusiasm among researchers for harnessing natural compounds in the development of pharmaceuticals has notably surged. Integrating phytomolecules with conventional antibiotics opens new vistas for discovering potent antimicrobial agents^26–28^. Phytochemicals often act through mechanisms distinct from conventional antibiotics, rendering them potentially valuable in combating resistant bacteria^26^. Several phytomolecules, such as gallic acid, curcumin, and thymol, have demonstrated antibacterial activity or synergistic effects with antibiotics, leading to a reduction in effective concentrations. Additionally, essential oils and their components have also been reported to enhance antibiotic efficacy when used in combination^15–17,24^.

**Fig. 1:**
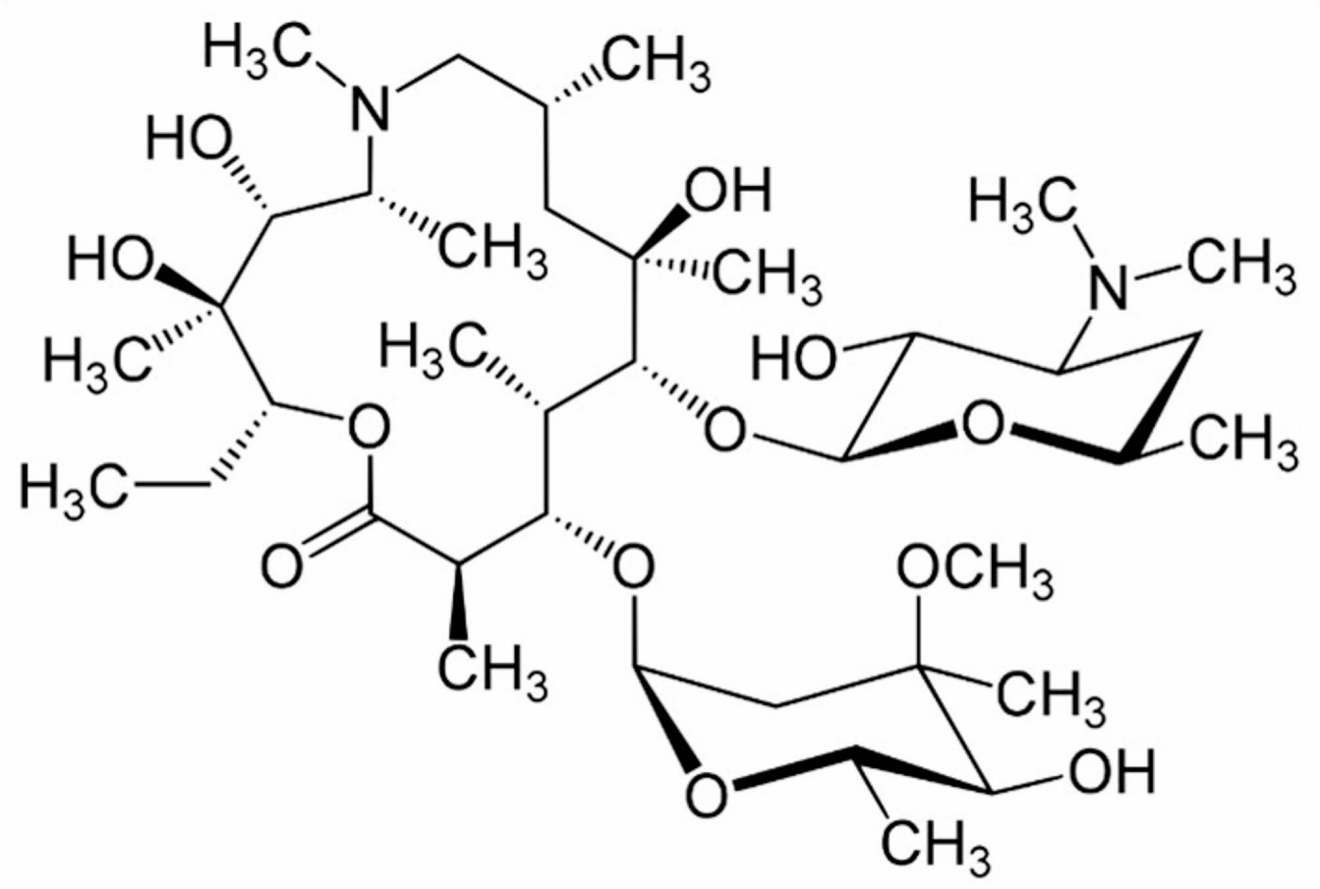
Chemical structures of azithromycin.

Our group has previously reported synergistic interactions between various phytomolecules, such as L-ascorbic acid, thymol, eugenol, berberine, quercetin, gallic acid, and curcumin, with antibiotics including erythromycin, ampicillin, amoxicillin, oxacillin, and azithromycin. One of our earlier studies demonstrated the mechanistic synergy between thymol and oxacillin against MRSA^15,16^. In continuation of this work, azithromycin was selected for the present study based on antibiotic susceptibility testing of the MRSA clinical isolates, where it exhibited high MIC values. This reduced susceptibility made azithromycin a strong candidate for synergy screening. Azithromycin, a macrolide antibiotic (Fig. 1), was selected for this study due to its widespread clinical use and known limitations against MRSA^29–31^. Despite its efficacy against various pathogens, azithromycin faces diminished activity against MRSA due to resistance mechanisms such as ribosomal methylation, efflux pump overexpression, and enzymatic degradation. These challenges limit its clinical effectiveness, making it a suitable candidate for evaluating potential synergy with phytochemicals. We chose to focus on azithromycin specifically, rather than other antibiotics, to investigate whether its effectiveness could be restored or enhanced when combined with a bioactive phytomolecule.

**Fig. 2:**
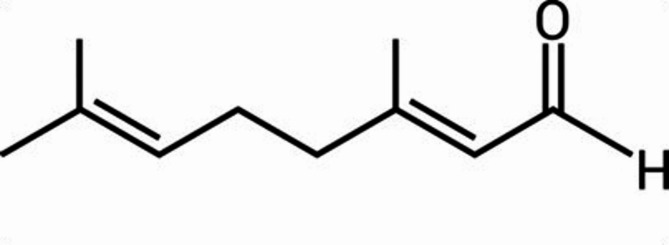
Chemical structures of citral.

In this context, citral, (3,7-dimethyl-2,6-octadienal), a monoterpene aldehyde found in essential oils (Fig. 2) from *Cymbopogon citratus*, *Litsea cubeba*, and *Melissa officinalis*, has garnered considerable attention for its potent antimicrobial, anti-inflammatory, and antioxidant properties. Prior studies has demonstrated antibacterial efficacy against various resistant pathogens, including MRSA, through multiple mechanisms. Prior studies indicate that citral inhibits bacterial efflux pumps, disrupts biofilm integrity, and enhances membrane permeability, thereby restoring the effectiveness of antibiotics^32–34^. While various phytomolecules have been studied for their antibacterial synergy, citral was specifically selected for this study based on its distinct multi-targeted mechanisms. Furthermore, citral is a GRAS-listed (Generally Recognized as Safe) compound with a favorable safety and pharmacokinetic profile, making it an ideal candidate for therapeutic evaluation. To the best of our knowledge, its synergistic potential in combination with azithromycin against MRSA remains underexplored, presenting an opportunity to investigate a novel and promising interaction.

In this study, we investigated the synergistic potential of citral in combination with azithromycin against MRSA clinical isolates. We assessed their in vitro bactericidal and bacteriostatic activities, PAE, and membrane-disrupting properties. Additionally, we performed scanning electron microscopy (SEM) to visualize structural damage in bacterial cells and conducted cytotoxicity assays to evaluate safety on human cells. This study provides critical insights into phytochemical-antibiotic synergy as a promising strategy for combating drug-resistant *S. aureus*, paving the way for future translational applications.

## Results and discussion

### Determination of MIC

In our previous research, we determined the antibiotic susceptibility of MRSA clinical isolates MB-0628, MB-0893, MB-1311, and MB-1137 to various antibiotics^1,5^ (Table S1 in Supplementary info). Building on these foundational results, our new findings provided insights into the efficacy of citral as an adjuvant to antibiotics. By evaluating citral’s MIC against these MRSA strains, we established a baseline for further investigation into its potential synergistic effects in combination with other antibiotics, specifically azithromycin. The following checkerboard assay investigates the interaction between citral and azithromycin to enhance antibacterial activity, emphasizing the potential of this combination therapy.

### Synergistic interaction between Citral and Azithromycin against MRSA clinical isolates

The synergistic potential of citral and azithromycin was rigorously evaluated using the checkerboard assay, revealing a marked enhancement in antibacterial efficacy against clinical isolates of *S. aureus*. The MICs of citral alone were found to be 250 µg/ml for MB-0628 and MB-1137, and 500 µg/ml for MB-0893 and MB-1311. The introduction of citral led to a substantial reduction in the MIC values of azithromycin, underscoring the powerful synergistic interaction between these two compounds.

The MIC of azithromycin alone ranged from 256 to 512 µg/ml across the tested strains. However, when combined with citral, the MIC values were dramatically reduced to 16 µg/ml, 64 µg/ml, 64 µg/ml, and 16 µg/ml for strains MB-0628, MB-0893, MB-1311, and MB-1137, respectively. This reduction corresponds to a 16-fold decrease in MB-0628 and MB-1137, and an 8-fold decrease in MB-0893 and MB-1311, highlighting the enhanced potency of azithromycin when paired with citral. Similarly, the MIC of citral alone, which varied between 250 and 500 µg/ml, was reduced to 15.6–62.5 µg/ml in combination with azithromycin, corresponding to a 16-fold decrease for MB-0628 and MB-1137 and an 8-fold decrease for MB-0893 and MB-1311. The calculated FICIs ranged from 0.093 to 0.249, consistently indicating a synergistic interaction (Table 1).

**Table 1:** Azithromycin with Citral *against clinical isolates of S. aureus*.

Strains	MIC in alone (µg/ml)		MIC of azithromycin-citral combination (µg/ml)				FICI	Interaction
	CIT	AZI	AZI	FR	CIT	FR		
**MB-0628**	250	256	16	16	15.6	16	0.124	Synergistic
**MB-0893**	500	512	64	8	62.5	8	0.249	Synergistic
**MB-1311**	500	512	64	8	62.5	8	0.249	Synergistic
**MB-1137**	250	512	16	32	15.6	16	0.093	Synergistic

*FICI, fractional inhibitory concentration index; FR, fold reduction; MIC, minimum inhibitory concentration; THY=Thymol; OXA=Oxacillin.

Notably, the combination of citral and azithromycin against strain MB-1137 demonstrated the lowest FICI (0.093), indicating an exceptionally strong synergistic effect. This pronounced synergy significantly reduced MIC values, underscoring the potential of this combination as a powerful therapeutic agent. Given these compelling results, the citral-azithromycin combination was selected for further in-depth studies. To explore its bactericidal and bacteriostatic properties, we conducted time-kill kinetics, aiming to gain deeper insights into its efficacy against resistant MRSA infections.

Notably, the combination of citral and azithromycin against strain MB-1137 demonstrated the lowest FICI (0.093), indicating an exceptionally strong synergistic effect. This pronounced synergy significantly reduced MIC values, underscoring the potential of this combination as a powerful therapeutic agent. The MIC of azithromycin in combination with citral was reduced to 16 µg/ml in MB0628 and MB1137 strains, which aligns with the susceptibility breakpoint, suggesting that this phytochemical–antibiotic pairing may help restore azithromycin’s activity against otherwise resistant strains. While these promising in vitro results provide a strong rationale for further investigation, future studies will be necessary to correlate these concentrations with achievable levels in vivo, taking into account pharmacokinetic and pharmacodynamic parameters.

### Time kill kinetics curve

**Fig. 3:**
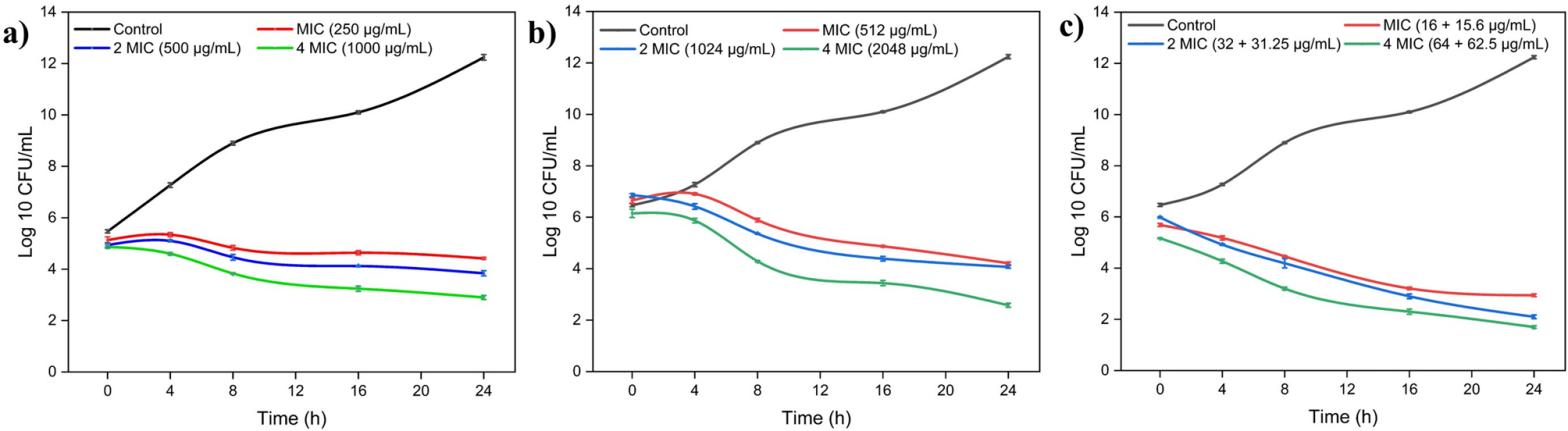
Time-kill curves for *Staphylococcus aureus* clinical isolate MB-1137 treated with (**a**) azithromycin, (**b**) citral, and (**c**) citral + azithromycin combination at MIC, 2×MIC, and 4×MIC concentrations over 24 h. Data represent mean ± SD (*n* = 3). Statistically significant differences between treatments at each time point were determined using one-way ANOVA followed by Tukey’s post hoc test (0 h: *p* = 1.33 × 10⁻⁶; 24 h: *p* = 1.93 × 10⁻¹⁴). The combination treatment showed significantly greater reduction in bacterial viability compared to individual treatments (*p* < 0.05). *“Note: The control CFU values observed at 24 h may reflect minor overestimation due to bacterial aggregation or clumping*,* which can artificially elevate viable counts in late log or early stationary phase cultures.”*.

The time-kill kinetics assay was conducted to assess the bacteriostatic/bactericidal effect of citral, azithromycin, and their combination against MRSA MB-1137 cells, using MIC, 2MIC, and 4MIC concentrations, with an untreated control. Cell viability was measured at 0, 4, 8, 16, and 24 h and expressed in log₁₀ CFU/ml (Table 2; Fig. 3).

**Table 2:** Cell viability of clinical isolate MB-1137 upon the exposure to Citral alone, Azithromycin alone and Citral + Azithromycin in combination at different MIC values.

Time (h)	Untreated control	Cell viability in log_10_ CFU/ml								
		Citral			Azithromycin			Citral + Azithromycin in combination		
		MIC	2MIC	4MIC	MIC	2MIC	4MIC	MIC	2MIC	4MIC
**0**	6.47	5.13	4.94	4.87	6.29	6.21	5.62	5.39	5.98	5.17
**4**	7.27	5.34	5.1	4.6	5.82	5.12	4.84	5.18	5.09	4.97
**8**	8.9	4.83	4.46	3.83	5.17	4.14	3.4	4.46	4.19	3.2
**16**	10.1	4.64	4.12	3.24	4.83	3.3	3.1	3.21	2.9	2.3
**24**	12.24	4.42	3.84	2.9	4.24	2.94	2.4	2.94	2.1	1.7

Citral, when used individually at its MIC (250 µg/ml), demonstrated a moderate reduction in cell viability, decreasing the bacterial count from an initial 5.13 log₁₀ CFU/ml to 4.42 log₁₀ CFU/ml after 24 h. Azithromycin (512 µg/ml) similarly reduced the viable count from 6.29 log₁₀ CFU/ml to 4.24 log₁₀ CFU/ml over 24 h, suggesting a concentration-dependent bacteriostatic effect for both agents. In contrast, the citral-azithromycin combination at MIC (15.6 µg/ml + 16 µg/ml) resulted in a more pronounced decline in bacterial count from 5.39 log₁₀ CFU/ml at 0 h to 2.94 log₁₀ CFU/ml at 24 h, indicating a bactericidal effect.

To support these observations, we performed one-way ANOVA followed by Tukey’s post hoc test using triplicate-simulated values based on the mean ± SD. Statistically significant differences were observed at both 0 h (*p* = 1.33 × 10⁻⁶) and 24 h (*p* = 1.93 × 10⁻¹⁴), confirming that the combination treatment was significantly more effective than individual treatments or the control. These findings reinforce the synergistic interaction of citral and azithromycin in reducing MRSA viability and validate the enhanced efficacy of combination therapy (Fig. 3).

### Post antibiotic effect

The PAE of azithromycin, citral, and their combination was evaluated against MRSA MB-1137 cells at MIC, 2MIC, and 4MIC concentrations. The PAE measures the duration of suppressed bacterial regrowth following exposure to antimicrobial agents, providing insights into their sustained efficacy. Azithromycin alone demonstrated a concentration-dependent PAE, ranging from 2.38 ± 0.08 h at MIC to 3.46 ± 0.06 h at 4MIC. Similarly, citral showed a slightly higher PAE, with values from 2.07 ± 0.11 h at MIC to 4.58 ± 0.06 h at 4MIC. Both agents exhibited primarily bacteriostatic effects, with limited ability to prevent bacterial regrowth over extended periods.

In contrast, the combination of citral and azithromycin produced a markedly enhanced PAE. At MIC, the combination resulted in a PAE of 4.26 ± 0.08 h, which extended significantly to 6.36 ± 0.07 h at 4MIC.

This extended suppression following removal of the antimicrobial agents highlights the enhanced pharmacodynamic potential of the citral–azithromycin combination. The significantly prolonged PAE suggests that the combination may delay bacterial regrowth more effectively than either agent alone, thereby reducing the likelihood of relapse and supporting more effective bacterial control post-exposure.

**Fig. 4:**
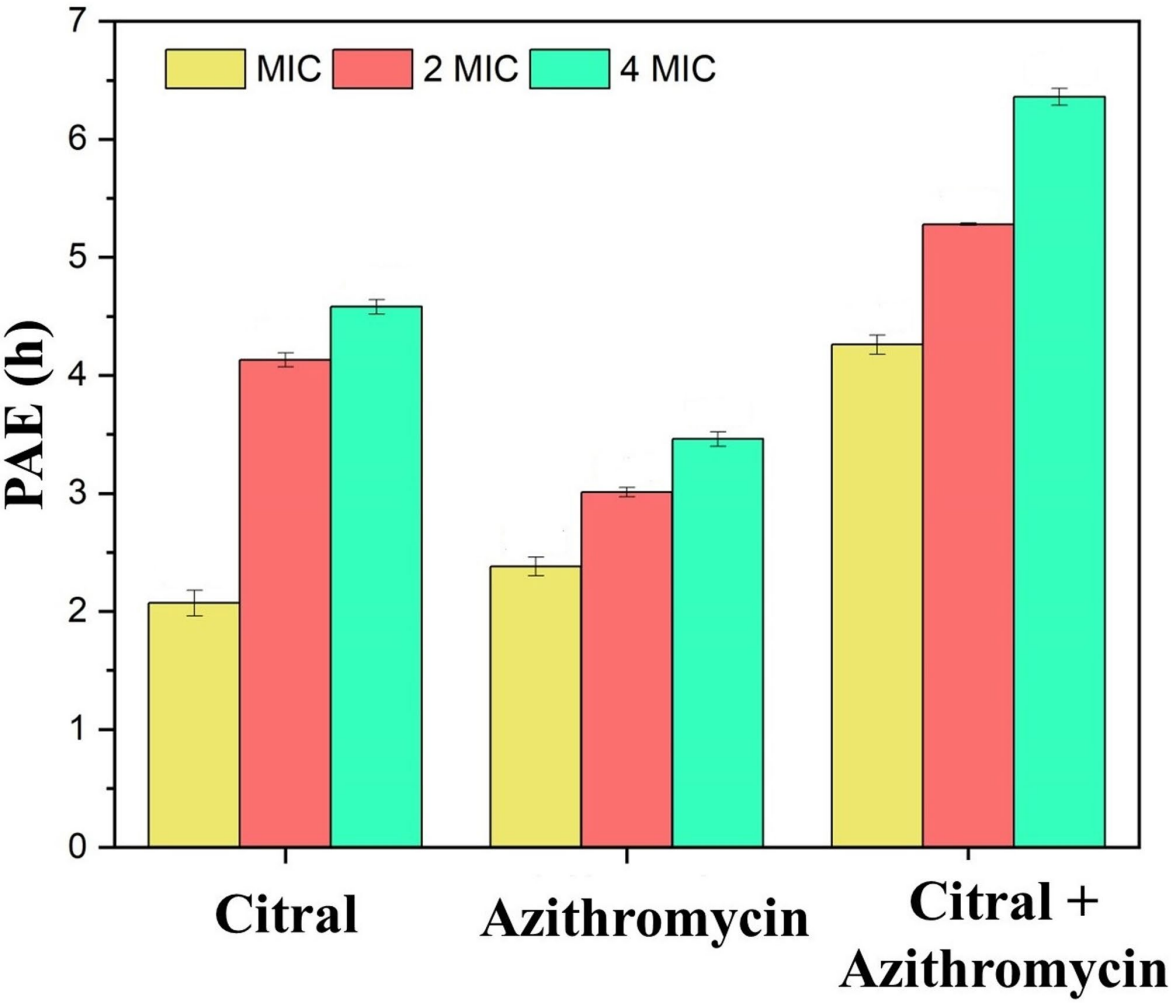
PAE duration of citral, azithromycin, and their combination against MRSA clinical isolate MB-1137. The duration of PAE (in hours) is evaluated for each treatment at MIC, 2×MIC, and 4×MIC.

This demonstrates the synergistic potential of citral and azithromycin in combination, offering both immediate bactericidal activity and extended post-antibiotic suppression. This dual effect is especially valuable in treating resistant MRSA infections, where prolonged bacterial suppression is crucial for preventing recurrence and resistance development (Table 3; Fig. 4).

**Table 3:** Comparison of PAE of citral, Azithromycin and Azithromycin-citral combination at MIC, 2MIC, 4MIC against MRSA clinical isolate MB-1137.

S. No.	Treatment	Mean PAE (h) ± SD		
		MIC	2 MIC	4 MIC
1.	Azithromycin	2.38 ± 0.08	3.01 ± 0.04	3.46 ± 0.06
2.	Citral	2.07 ± 0.11	4.13 ± 0.06	4.58 ± 0.06
3.	Citral + Azithromycin	4.26 ± 0.08	5.28 ± 0.01	6.36 ± 0.07

### SEM analysis

**Fig. 5:**
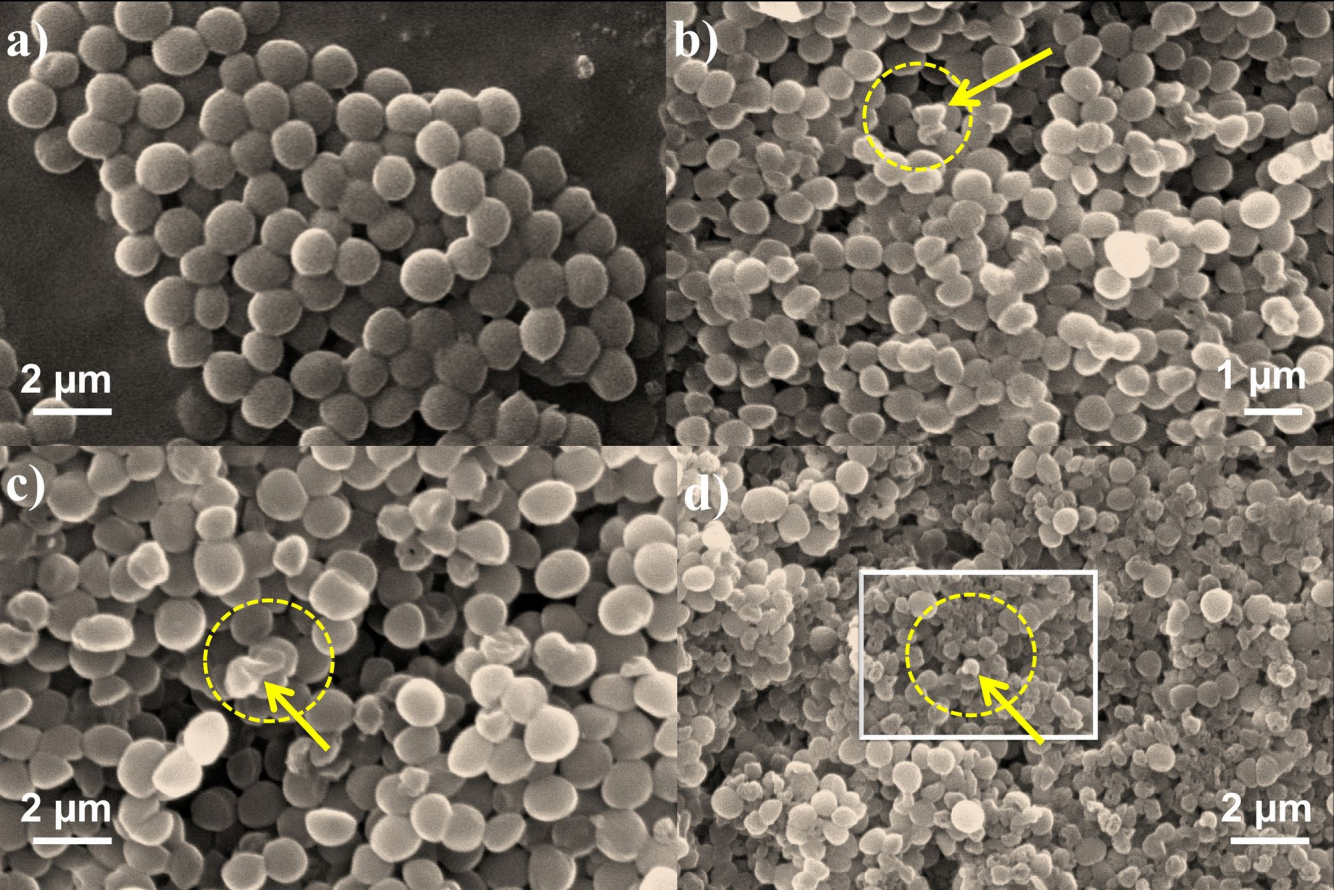
SEM images of MRSA clinical isolate MB-1137. (**a**) Untreated control cells exhibiting normal morphology; (**b**) Cells treated with citral, demonstrating structural alterations indicative of membrane disruption; (**c**) Cells treated with azithromycin, showing characteristic changes associated with antibiotic action; (**d**) Cells treated with the citral-azithromycin combination at ½ MIC concentration, revealing pronounced morphological damage. These images illustrate the differential effects of treatments on bacterial cell integrity, highlighting the potential synergistic action of the citral-azithromycin combination.

SEM analysis was performed to investigate the morphological changes in MRSA strain MB-1137 upon treatment with ½ MIC concentrations of citral, azithromycin, and their combination. To visualize early-stage morphological alterations without inducing complete cell lysis, ½ MIC concentrations were intentionally selected. The control group (Fig. 5a) exhibited well-defined, intact bacterial cells with smooth surfaces, characteristic of untreated MRSA. Upon treatment with citral at 125 µg/ml (Fig. 5b), the bacterial cells showed slight surface irregularities and mild structural damage, indicating initial disruption of cell membrane integrity. Azithromycin treatment at 256 µg/ml (Fig. 5c) led to more pronounced morphological changes, including deformation and shrinkage of bacterial cells, reflecting its inhibitory action on protein synthesis.

However, the most significant disruption was observed with the combination of citral (15.6 µg/ml) and azithromycin (32 µg/ml) (Fig. 5d). This group demonstrated severe cellular disintegration, with many cells appearing collapsed, fragmented, and exhibiting marked membrane damage. The pronounced structural alterations suggest a synergistic mechanism, potentially driven by citral’s membrane-targeting effects coupled with azithromycin’s inhibition of bacterial protein synthesis. These observations correlate with the synergistic reduction in MIC values reported in the checkerboard assay, underscoring the enhanced efficacy of the combination therapy, even at sub-MIC concentrations.

### Membrane damaging potential

#### The nucleic acid release

**Fig. 6:**
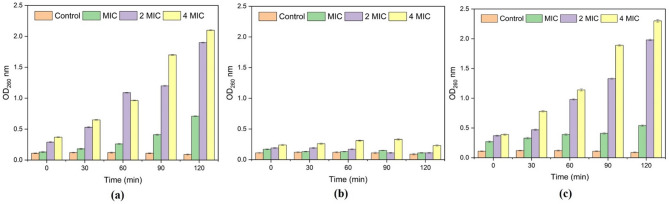
Assessment of nucleic acid content release from MRSA clinical isolate MB-1137. The graph depicts the release of intracellular components following treatment with (**a**) citral alone, (**b**) azithromycin alone, and (**c**) the combination of citral with azithromycin. Measurements were taken at minimum inhibitory concentration (MIC), 2×MIC, and 4×MIC. Data are presented as mean values with standard deviations, highlighting the effects of each treatment on cellular integrity.

The PAE of citral, azithromycin, and their combination was evaluated against MRSA clinical isolate MB-1137 at MIC, 2×MIC, and 4×MIC concentrations. The PAE measures the time delay in bacterial regrowth following a brief exposure to antimicrobial agents, offering insights into the duration of suppressed bacterial recovery once the agent is removed. Citral exhibited a concentration-dependent PAE, ranging from 2.07 ± 0.11 h at MIC to 4.58 ± 0.06 h at 4×MIC, suggesting its intrinsic potential to prolong bacterial regrowth suppression even as a standalone agent (Fig. 6a). Azithromycin, when used alone, also showed a PAE increasing with concentration, from 2.38 ± 0.08 h at MIC to 3.46 ± 0.06 h at 4×MIC, indicating modest post-antibiotic effects (Fig. 6b). In comparison, the citral–azithromycin combination demonstrated the most pronounced post-antibiotic suppression, with a PAE of 4.26 ± 0.08 h at MIC and a significantly extended value of 6.36 ± 0.07 h at 4×MIC, clearly outperforming either compound alone (Fig. 6c).

This prolonged post-antibiotic effect reflects a delayed recovery of bacterial growth after drug withdrawal, which is distinct from the bactericidal effect observed in the time-kill kinetics assay where drugs are present throughout the incubation period. The enhanced PAE in the combination group suggests that citral may contribute to sustained metabolic disruption, enhancing azithromycin’s suppressive activity even after removal of the drugs. This dual mechanism, involving immediate bactericidal effect and prolonged post-antibiotic suppression, may be especially beneficial in treating resistant MRSA infections, where preventing regrowth is crucial to avoiding recurrence and resistance development.

#### Bacterial protein leakage by citral-azithromycin combination

**Fig. 7:**
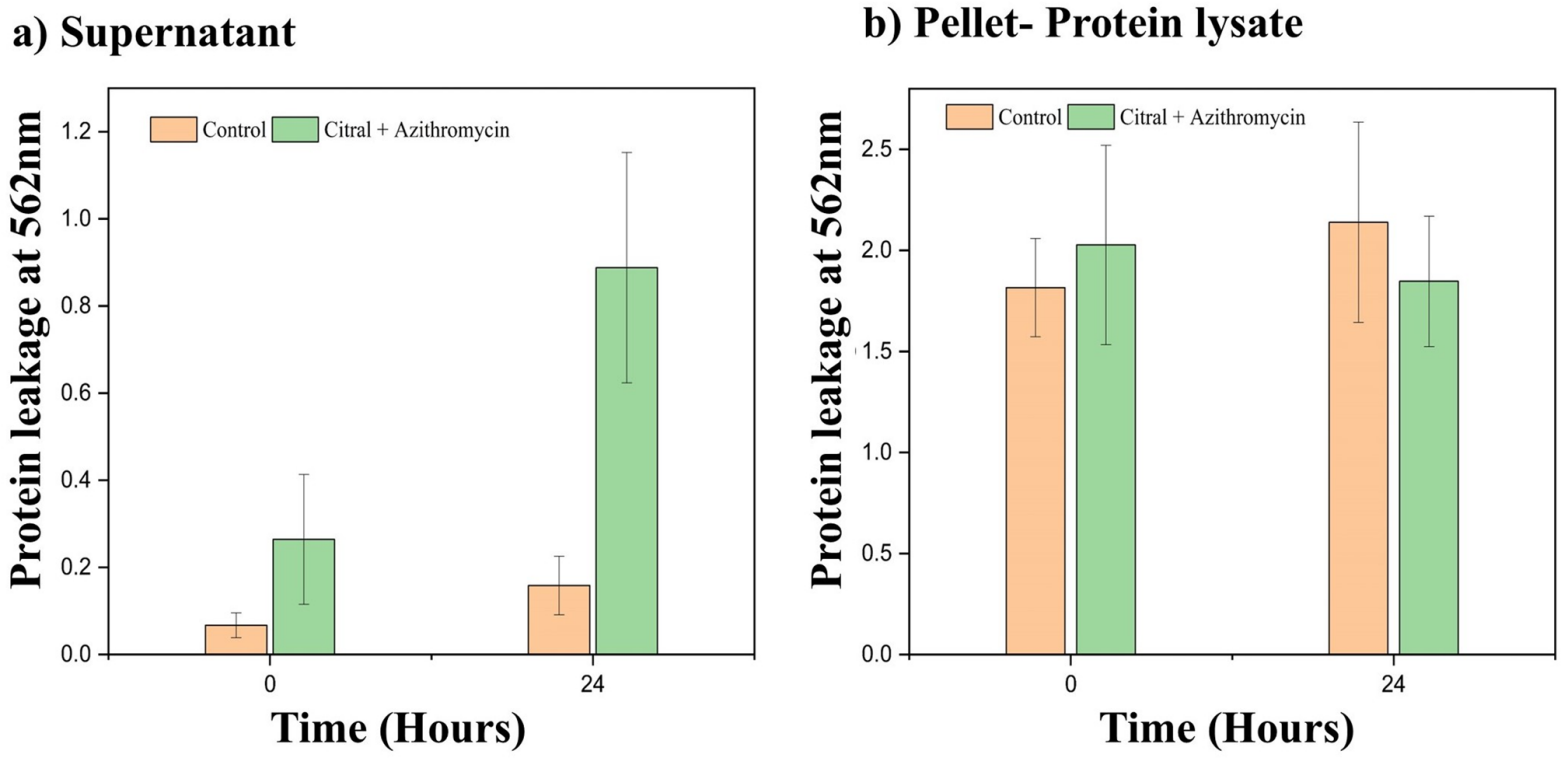
Protein Leakage Assay of MRSA Clinical Isolate MB-1137 Treated with Citral-Azithromycin Combination at MIC Concentration. (**a**) Supernatant and (**b**) Pellet cell lysate collected at 0 h and 24 h from untreated control and citral-azithromycin treated samples.

To evaluate the impact of the citral-azithromycin combination on bacterial cell integrity, we measured the protein release into the supernatant and pellet fractions following treatment (Fig. 7a and b). The results demonstrate the extent of cell membrane damage inflicted by the citral-azithromycin combination at MIC concentration over a 24-hour period. The comparison between treated and untreated samples highlights a significant rise in extracellular protein levels in the supernatant for the treated cells. Specifically, at 24 h, the protein concentration in the supernatant from treated cells increased by approximately 2.5-fold compared to the untreated control, suggesting that the treatment induces membrane disruption and subsequent release of intracellular contents.

In the pellet cell lysate, a notable reduction in protein levels was observed after 24 h, reflecting potential cellular lysis and diminished structural integrity, corroborating the increased supernatant protein content. These observations support the hypothesis that citral and azithromycin act synergistically to compromise bacterial cell membranes, leading to extensive protein leakage into the extracellular space. Such outcomes emphasize the combination’s capacity to disrupt MRSA cellular integrity, pointing to its potential as an effective therapeutic strategy against resistant pathogens.

### Safety studies

**Fig. 8:**
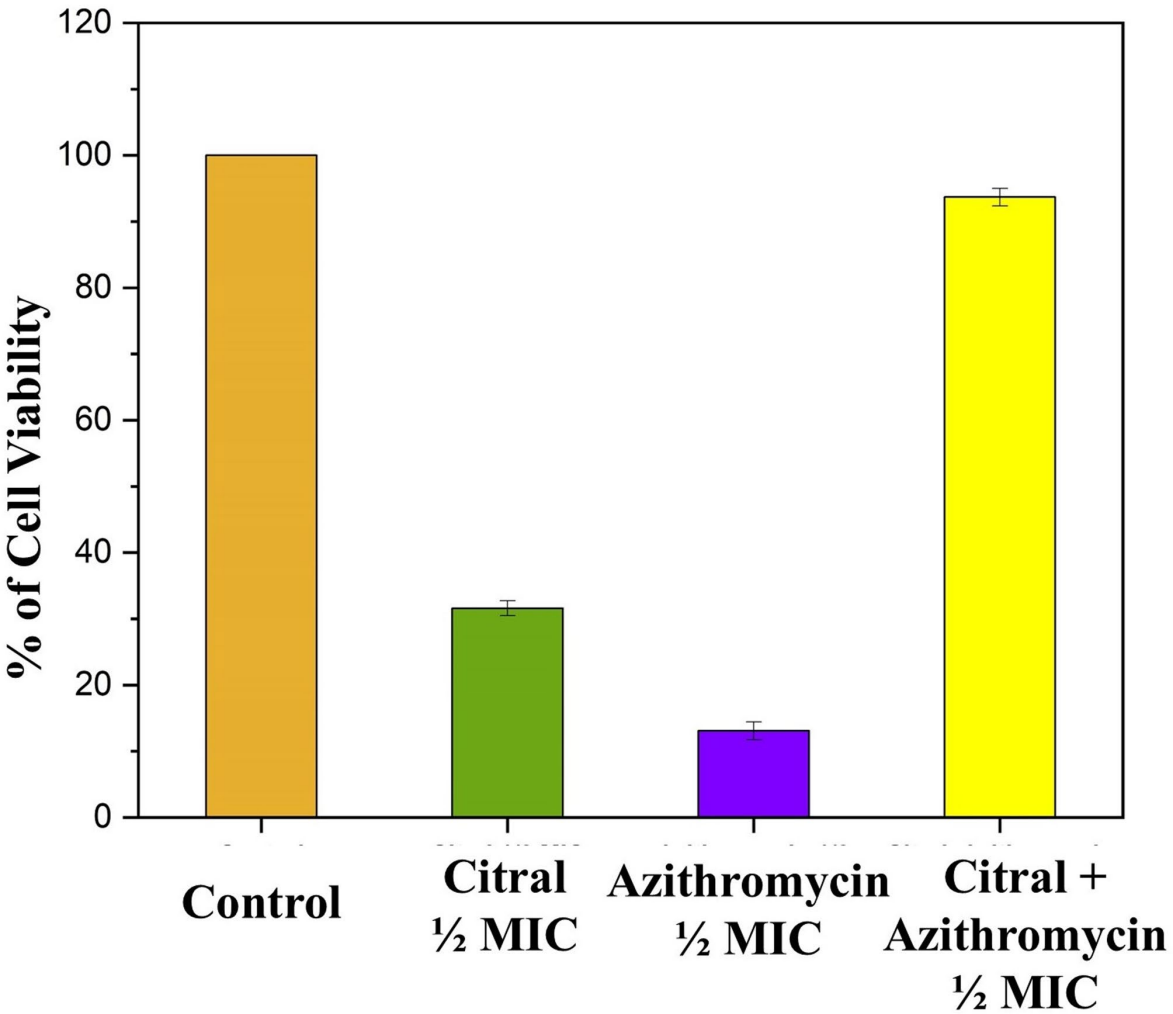
Cell viability percentage of BJ1-hTERT cells treated with citral (125 µg/ml), azithromycin (256 µg/ml), and their combination (7.8 µg/ml + 8 µg/ml**)**. Staining with crystal violet was used to assess cytotoxicity after 24-hour exposure. Data are represented as mean ± SD (*n* = 3).

The cytotoxicity assay on BJ1-hTERT cells revealed that citral and azithromycin, when used individually, significantly reduced cell viability, indicating a considerable cytotoxic effect (Fig. 8). Specifically, citral alone at 125 µg/ml concentration resulted in a cell viability of 31.60%, while azithromycin at 256 µg/ml concentration further reduced viability to 13.08%. These findings highlight the cytotoxic effects of each compound when administered separately. These concentrations correspond to ½×MICs, which were taken to avoid solubility issues observed at MIC and to ensure the evaluation of physiologically relevant, non-toxic doses. In contrast, the combination of citral-azithromycin (7.8 µg/ml + 8 µg/ml) demonstrated negligible adverse effect, reaching 93.7% cellular viability. This suggests a potential protective or mitigating effect when both agents are used together at lower concentrations, which not only significantly alleviates cytotoxicity but also supports cell survival. These results illustrate the therapeutic advantage of using the citral-azithromycin combination, which may enhance safety profiles by reducing cytotoxic effects on human cells compared to single-agent treatments, thereby highlighting its promise as a more biocompatible treatment option.

## Materials and methods

### Phytomolecules and antibiotics

Citral (purity ≥ 98%) was procured from Thermo Fisher Scientific, while azithromycin (purity ≥ 99%) was obtained from TCI (India) Pvt. Ltd.

### Bacterial strains used in the study

Clinical isolates of *S. aureus* (MB-0628, MB-0893, MB-1311, and 1137) were obtained from St. Stephen’s Hospital, New Delhi, India. Their resistance to commonly used antibiotics was assessed according to the Clinical and Laboratory Standards Institute (CLSI) guidelines. The *S. aureus* strain MTCC-96 was obtained from CSIR-IMTECH, Chandigarh, India, while the ATCC-43300 strain was procured from KWIK-STIK™.

### Human cells

BJ1-hTERT normal human immortalized fibroblasts, derived from foreskin, were utilized for in vitro testing of selected citral, azithromycin, and their combinations. These cells were cultured in Dulbecco’s Modified Eagle Medium (DMEM) from Himedia Laboratories, supplemented with 10% (v/v) fetal bovine serum (Gibco), 100 U/ml penicillin, 0.1 mg/ml streptomycin, and 2 mM glutamine. Mycoplasma contamination was ruled out by performing DAPI staining^35^.

### Broth microdilution method

Minimum inhibitory concentrations (MICs) were determined using Mueller-Hinton broth (MHB) in 96-well microtiter plates, in accordance with CLSI guidelines (CLSI 2020). Citral and azithromycin were serially diluted and tested against MTCC-96, ATCC-43,300, and various clinical isolates. The assay was performed with an initial bacterial inoculum of 5 × 10^5^ CFU/ml. The columns 11 and 12 of the microtiter plate served as positive controls for bacterial growth and sterility, respectively. A 10 µL aliquot of the bacterial suspension was added to each well, excluding the negative control. The plates were incubated at 37 °C for 16–24 h. MIC values were determined based on the absence of turbidity, which was interpreted as MIC. To confirm microbial viability, *p*-iodonitrotetrazolium violet (INT) dye (0.2 mg/ml) was added to each well. The experiment was conducted in triplicate, and average MIC values were calculated^16,17,35,36^.

### Interactions between Citral and Azithromycin (Checkerboard Assay)

The interactions between citral and azithromycin against clinical isolates of *S. aureus* were evaluated using the checkerboard assay, conducted in triplicate for accuracy. The synergy between citral and azithromycin was assessed quantitatively through fold reduction and the fractional inhibitory concentration index (FICI). The FICI values were summed to determine the overall FIC index, which indicates the type of interaction: synergy (FICI ≤ 0.5), additivity (FICI > 4.0), or no interaction (antagonism, FICI > 0.5–4.0)^41^. The FICI was calculated using the following equation:

$$\:FIC\:\left(Phytomolecule\right)=\frac{MIC\:of\:phytomolecule\:in\:combination}{MIC\:of\:phytomolecule\:\:alone}$$

$$\:FIC\left(Antibiotic\right)=\frac{MIC\:of\:antibiotic\:in\:combination}{MIC\:of\:antibiotic\:alone}$$

$$\:FIC\:Index\:\left(FICI\right)=FIC\:\left(Phytomolecule\right)+FIC\left(Antibiotic\right)$$

### Time-Kill assay (Bactericidal/Bacteriostatic activity)

The in vitro bactericidal and bacteriostatic effects of citral, azithromycin, and their combination on the clinical isolate MB-1137 were assessed following our earlier reported procedure. Bacterial cultures were incubated in Mueller-Hinton broth (MHB) at 37 °C for 24 h. The suspension’s turbidity was standardized to 0.5 McFarland (10^7^ CFU/ml) using sterile normal saline. A 200 µL aliquot of this suspension was then introduced into 20 ml of MHB within conical flasks containing citral, azithromycin, and combination of both at concentrations equivalent to MIC, 2×MIC, and 4×MIC. A control inoculum with 1% DMSO was also prepared. The cultures were incubated at 37 °C, and samples (100 µL) were collected at 0, 4, 8, 16, and 24 h. These cultures were then plated on Mueller-Hinton agar (MHA) to determine viable cell counts^38–40^. All experiments were performed in triplicate, with appropriate untreated controls. Time-kill curves were plotted as log₁₀ CFU/mL versus time (hours). Statistical analysis was performed at 0 and 24 h using one-way ANOVA followed by Tukey’s HSD post-hoc test to evaluate significant differences between treatment groups. Data are expressed as mean ± standard deviation (*n* = 3), and a p-value of < 0.05 was considered statistically significant.

### Post-Antibiotic effect

PAE was evaluated using the viable count method as described earlier by us. In summary, an overnight culture of the MRSA clinical isolate MB-1137 was diluted in MHB to achieve a bacterial count of approximately 10^7^ CFU/ml. This bacterial suspension was subsequently treated with citral, azithromycin and their combination at MIC, 2×MIC, and 4×MIC concentrations for 2 h at 37 °C. Following exposure, the antibiotics were removed by centrifugation and subsequent washing with phosphate-buffered saline (PBS). The bacterial pellets were resuspended in fresh MHB and incubated at 37 °C. Samples were taken at 0, 2, 4, 6, and 8 h post-antibiotic removal and plated on MHA plates. The plates were incubated at 37 °C for 24 h, and the resulting colonies were counted. The PAE was determined by calculating the difference in time taken by the treated and untreated control bacteria to reach the same log_10_ CFU/ml. The PAE results were reported as the mean ± standard deviation (SD) from three independent experiments^35,41^.

### Bacterial morphology examination using scanning Electron microscopy (SEM)

SEM was employed to analyze morphological changes in bacterial cells after following treatment with citral, azithromycin, and their combination. The sample preparation followed the procedure previously described by us^35^. Bacterial cells in the mid-exponential growth phase were treated with citral and azithromycin at their respective MICs and incubated at 37 °C for 24 h. After incubation, both treated and control samples were centrifuged at 10,000 rpm for 15 min. The collected cell pellets were washed three times with PBS and initially fixed with 2.5%glutaraldehyde at 4 °C for 6 h, followed by secondary fixation with 1% osmium tetroxide for 1 h. The fixed samples were then dehydrated using a series of graded ethanol concentrations (25% 50% 70% 80% 90% 95%and 100% prior to SEM analysis^35,40^.

### Evaluation of membrane damage

#### Measurement of nucleic acid release

Cell membrane disruption often leads to the release of small molecules such as purines, pyrimidines, and amino acids. Membrane damage was quantified by measuring the absorbance at 260 nm, which reflects the presence of these compounds. In summary, bacterial cultures were grown in 50 ml of MHB at 37 °C, then standardized to achieve an approximate inoculum of 10^6^ CFU/ml. Treatments were administered using citral, azithromycin, and a citral- azithromycin combination at concentrations of MIC, 2×MIC, and 4×MIC. Samples were incubated at 37 °C and collected at 0, 30, 90, and 120 min. Absorbance at 260 nm was then measured to assess membrane permeability alterations. After incubation, cells were centrifuged at 5,000 g for 10 min, then resuspended in 0.85% w/v saline solution. Each experiment was performed in triplicate with independently grown cultures, and the results were presented as mean ± standard deviation^42^.

#### Assessment of protein leakage

To further evaluate cell integrity, the release of proteins from bacterial cells into the supernatant was measured. Overnight bacterial cultures were treated with the citral-azithromycin combination at MIC levels. Samples of both the bacterial pellets and supernatants were collected at baseline and after 24 h of treatment. Pelleted cells were lysed using 50 µL of buffer containing 2% SDS, 0.375 M Tris (pH 6.8), and 3.4 M sucrose, with added protease inhibitors. The mixture was incubated at 100 °C for 6 min. Following centrifugation at 14,000 rpm for 25 min, the supernatant containing the proteins was isolated and stored at − 20 °C until further use. Protein concentrations for both intracellular and extracellular samples were determined using the bicinchoninic acid (BCA) assay, according to the manufacturer’s protocol. Standard curves were generated with bovine serum albumin (BSA) to quantify the protein concentration by absorbance at 562 nm^35^.

### Safety studies

BJ1-hTERT cells were plated in 96-well plates at a density of 10,000 cells per well in complete growth medium. When cells reached 30–40% confluency, they were treated with ½ MIC concentrations of the test compounds and antibiotics for 24 h. Control wells received only the vehicle (1% DMSO). After the 24 h treatment period, the plates were washed twice with 1× phosphate-buffered saline (PBS), followed by staining with a 0.5% crystal violet solution (SRL Chemicals) for 20 min at 25^o^C on an orbital rocker set to 200 rpm. The plates were then rinsed 3–4 times with distilled water and allowed to air dry overnight at 25^o^C. To solubilize the stain, 100 µL of methanol was added to each well, and the plates were incubated with lids at 25^o^C for 20 min. Optical density (OD) was measured at 570 nm using a microplate reader (Thermo Fisher Scientific). Cell viability was determined by calculating the background-subtracted absorbance of the treated wells relative to the control wells using the following formula:

$$\text{Cell}\: \text{viability} (\%) = (\text{Absorbance}\:\text{of}\:\text {treated}\:\text {well}/\text{Absorbance}\:\text{of}\:\text{control}\:\text{ well}) \times 100.$$

The data was presented as the mean ± standard deviation (SD) from three independent experiments^35,43,44^.

## Conclusion

The escalating threat of multidrug-resistant MRSA necessitates innovative therapeutic strategies to combat its persistent resistance. Our study demonstrates that the citral-azithromycin combination offers a dual-mechanism, synergistic approach, combining citral’s membrane disrupting activity with azithromycin’s ribosomal inhibition to achieve potent bactericidal effects against clinical MRSA isolates. This combination not only reduces MIC values by 16–32 fold but also extends PAE to 4.26 h, significantly delaying bacterial regrowth. Furthermore, the combination exhibits a favorable safety profile, with 93.7% cell viability in human fibroblasts, highlighting its potential for clinical translation. By addressing the dual challenges of efficacy and cytotoxicity, this innovative strategy fills a critical gap in MRSA treatment and paves the way for future development of phytochemical-antibiotic therapies against multidrug-resistant pathogens.

## Electronic supplementary material

Below is the link to the electronic supplementary material.

Supplementary Material 1

## Data Availability

The datasets used and/or analyzed during the current study are available from the corresponding author on reasonable request.
