# Supplementary material for: Counteracting methicillin resistant Staphylococcus aureus through novel Citral-Azithromycin combination
Source: Sci Rep. 2025 Jul 15;15:25548. doi: 10.1038/s41598-025-11721-4 (PMC12263860; doi:10.1038/s41598-025-11721-4)
Supplement: Supplementary file 1 — Supplementary Material 1 [file 41598_2025_11721_MOESM1_ESM.docx]

**Counteracting Methicillin Resistant *Staphylococcus aureus* through Novel Citral-Azithromycin Combination**

**Hitesh K Sharma^1^, Ibha Singh^2^, Amarnath Karna^2^, Puneet Gupta^1^, Taru Singh^3^, Anoop Kumar^4^, Deepti Pandita^5^, Monalisa Mukherjee^6^, Virinder S Parmar^6, 7^, Pallavi Agarwal^2^, Viney Lather^1^***

*^1^Amity Institute of Pharmacy, Amity University Uttar Pradesh, Sector 125, Noida 201 313, India*

*^2^Amity Institute of Molecular Medicine and Stem Cell Research, Amity University Uttar*

*Pradesh, Sector 125, Noida 201 313, India*

*^3^Amity Institute of Biotechnology, Amity University, Noida*

*^4^Department of Pharmacology, Delhi Pharmacological Sciences and Research University (DPSRU), New Delhi, 110 017, India*

*^5^Department of Pharmaceutics, Delhi Institute of Pharmaceutical Sciences & Research (DIPSAR) Delhi Pharmaceutical Sciences and Research University, Pushp Vihar, Government of NCT of Delhi, New Delhi 110 017, India*

*^6^Amity Institute of Click Chemistry and Research Studies, Amity University Uttar Pradesh, Sector 125, Noida 201 313, India*

*^7^Nanoscience Program, CUNY Graduate Center and Department of Chemistry and Environmental Science, Medgar Evers College, The City University of New York, 1638 Bedford Avenue, NY 10025, USA*

***Corresponding Author:** Dr. Viney Lather

**Correspondence to:** Dr. Viney Lather, Amity Institute of Pharmacy, Amity University Uttar Pradesh, Sector 125, Noida 201 313, India,

**Email:** [vlather@amity.edu](mailto:vlather@amity.edu)

**Table S1:** Antibiotic sensitivity profiling against Clinical isolates of *Staphylococcus aureus.*

| **S. No.** | **Antibiotics** | **MIC in (µg/mL)** | | | | | |
| --- | --- | --- | --- | --- | --- | --- | --- |
|  |  | **MTCC-96** | **ATCC-43300** | **MB0628** | **MB0893** | **MB1311** | **MB1137** |
| **1** | **Ampicillin** | 0.19 | 64 | 256 | 512 | 256 | 128 |
| **2** | **Amoxicillin** | 0.78 | 128 | 256 | 512 | 256 | 128 |
| **3** | **Oxacillin** | 0.39 | 64 | 128 | 256 | 256 | 128 |
| **4** | **Penicillin** | 1.56 | 512 | 512 | 1024 | 512 | 512 |
| **5** | **Cefoxitin** | 0.039 | 64 | 128 | 128 | 64 | 64 |
| **6** | **Ceftriaxone** | 0.19 | 7.8 | 64 | 128 | 128 | 128 |
| **7** | **Ciprofloxacin** | 0.19 | 4 | 16 | 32 | 16 | 8 |
| **8** | **Levofloxacin** | 0.09 | 8 | 4 | 8 | 4 | 8 |
| **9** | **Moxifloxacin** | 0.04 | 2 | 2 | 2 | 1 | 1 |
| **10** | **Ofloxacin** | 0.39 | 16 | 16 | 16 | 8 | 16 |
| **11** | **Erythromycin** | 0.78 | 1024 | 1024 | 1024 | 512 | 1024 |
| **12** | **Azithromycin** | 0.39 | 512 | 256 | 512 | 512 | 512 |
| **13** | **Linezolid** | 2 | 2 | 2 | 4 | 4 | 4 |
| **14** | **Daptomycin** | 0.25 | 1 | 1 | 0.5 | 0.5 | 1 |
| **15** | **Tetracycline** | 0.39 | 4 | 2 | 16 | 2 | 4 |
| **16** | **Vancomycin** | 0.5 | 1 | 2 | 1 | 1 | 2 |
